# FlyBase portals to human disease research using *Drosophila* models

**DOI:** 10.1242/dmm.023317

**Published:** 2016-03-01

**Authors:** Gillian H. Millburn, Madeline A. Crosby, L. Sian Gramates, Susan Tweedie, the FlyBase Consortium

**Affiliations:** 1 Department of Genetics, University of Cambridge, Downing Street, Cambridge CB2 3EH, UK; 2 The Biological Laboratories, Harvard University, 16 Divinity Avenue, Cambridge, MA 02138, USA

**Keywords:** *Drosophila*, Disease model, Online resource, FlyBase

## Abstract

The use of *Drosophila melanogaster* as a model for studying human disease is well established, reflected by the steady increase in both the number and proportion of fly papers describing human disease models in recent years. In this article, we highlight recent efforts to improve the availability and accessibility of the disease model information in FlyBase (http://flybase.org), the model organism database for *Drosophila*. FlyBase has recently introduced Human Disease Model Reports, each of which presents background information on a specific disease, a tabulation of related disease subtypes, and summaries of experimental data and results using fruit flies. Integrated presentations of relevant data and reagents described in other sections of FlyBase are incorporated into these reports, which are specifically designed to be accessible to non-fly researchers in order to promote collaboration across model organism communities working in translational science. Another key component of disease model information in FlyBase is that data are collected in a consistent format ­­– using the evolving Disease Ontology (an open-source standardized ontology for human-disease-associated biomedical data) – to allow robust and intuitive searches. To facilitate this, FlyBase has developed a dedicated tool for querying and navigating relevant data, which include mutations that model a disease and any associated interacting modifiers. In this article, we describe how data related to fly models of human disease are presented in individual Gene Reports and in the Human Disease Model Reports. Finally, we discuss search strategies and new query tools that are available to access the disease model data in FlyBase.

## Abstract (precis)

***Drosophila* Collection:** *Drosophila melanogaster* is well established as a model for studying human disease. Here, we highlight recent efforts to enhance the availability and accessibility of disease model data in FlyBase, the model organism database for *Drosophila*.

## Introduction

*Drosophila melanogaster* research has incorporated models of human disease for more than two decades [for example, xeroderma pigmentosum (Mounkes et al., 1992); amyotrophic lateral sclerosis (Phillips et al., 1995); Machado-Joseph disease (Warrick et al., 1998); Huntington disease (Jackson et al., 1998); Parkinson disease (Feany and Bender, 2000); neurodegenerative diseases (Jaiswal et al., 2012)]. The number of research papers describing the development or use of fly models of disease has been increasing steadily, from fewer than 40 papers in the year 2000 (less than 2% of total fly papers published that year) to almost 300 papers in 2014 (11% of total fly papers). This large body of work provides a valuable resource both for *Drosophila* researchers interested in human health models, and for clinical researchers who would like to explore existing disease models in flies or who would like to exploit fly models to study their disease of interest.

Since 1992, FlyBase (http://flybase.org), the model organism database for *Drosophila* (dos Santos et al., 2015)*,* has compiled genetic and genomic information from the research literature and from high-throughput data sources, providing an extensive online resource. One of the key advantages of working with *Drosophila melanogaster* is the large number of genome-scale reagent collections that have been created by many groups and made available in public repositories (reviewed in Mohr et al., 2014). These include an extensive complementary DNA (cDNA) resource (Stapleton et al., 2002), multiple insertional mutagenesis collections (Bellen et al., 2004, 2011), comprehensive RNA interference (RNAi) collections for targeted gene knockdown in cells (Boutros et al., 2004; Flockhart et al., 2012) or in flies (for example, Dietzl et al., 2007; Ni et al., 2011), and an expanding set of protein trap collections (Morin et al., 2001; Buszczak et al., 2007; Nagarkar-Jaiswal et al., 2015). A wide range of sophisticated molecular techniques are available to engineer the *Drosophila* genome (reviewed in Ejsmont and Hassan, 2014; Bassett and Liu, 2014; Beumer and Carroll, 2014), including the CRISPR/Cas9 system (Bassett et al., 2013; Gratz et al., 2013; Kondo and Ueda, 2013; Ren et al., 2013; Sebo et al., 2014; Yu et al., 2013). This means that appropriate mutations can be relatively easily engineered. FlyBase includes descriptions of available reagents and provides links to the public repositories that distribute them. One of the sections of the Human Disease Model Report (reports from FlyBase that provide background information on a specific disease, a tabulation of related disease subtypes, and summaries of experimental data and results using *Drosophila melanogaster*) is designed to facilitate access to useful reagents relevant to a specific disease model (described below).

Reflecting the strength of *Drosophila* as a genetic research organism, FlyBase data organization revolves around genes. This gene-centric approach has served *Drosophila* researchers well, but often does not provide an intuitive entry point into the database for other researchers. It can also limit the presentation of data that encompasses many genes or complex aspects of developmental and cellular biology, including models of human disease. Here, we describe a two-pronged approach, one disease-centric and the other gene-centric, that FlyBase has pursued to address the need to make *Drosophila* translational research more accessible and visible to a wider community. FlyBase has recently introduced a new report format, the Human Disease Model Report, which is designed to provide a generally accessible entry point into the database for all researchers interested in human disease and to provide an integrated view of research involving *Drosophila* models of human disease. In addition, Gene Reports have been expanded to include compilations of data related to models of human disease, captured in a rigorous and easily searchable format. We conclude with a discussion of search and browsing options, including a new dedicated tool for disease-related queries.

## An overview of FlyBase Human Disease Model Reports: ALS as an example

**Fig. 1.**
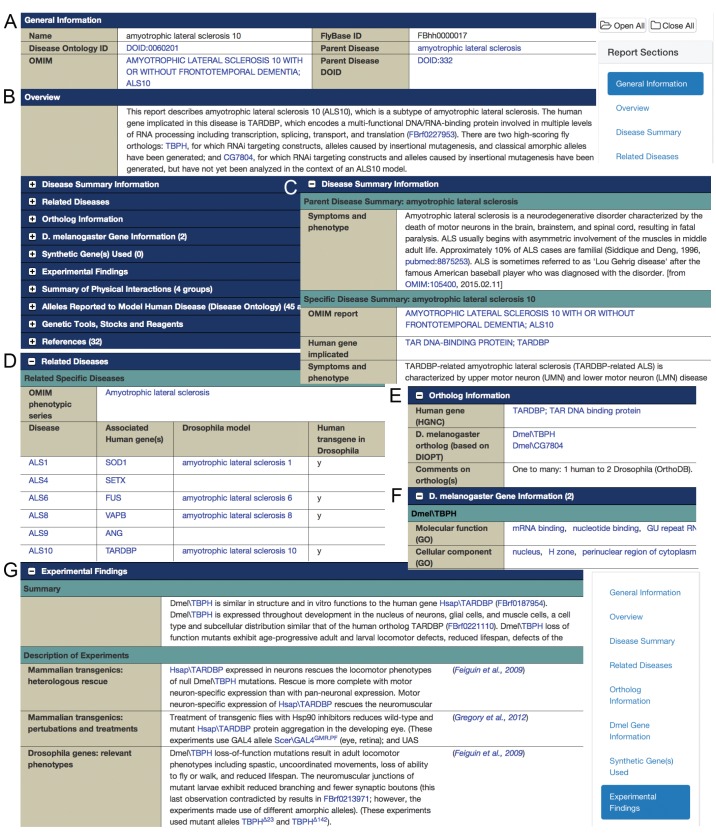
**The Human Disease Model Report for amyotrophic lateral sclerosis 10 (ALS10).** Selected sections of the report, as it appeared in September 2015, are shown. (A) General Information; (B) Overview; (C) Disease Summary Information; (D) Related Diseases; (E) Ortholog Information; (F) D. melanogaster Gene Information; and (G) Experimental Findings. Major section headings are indicated on the dark blue bars and in the floating ‘Report Sections’ panel (top right and bottom right). Initially, most sections are closed (as indicated by the ‘+’ icons in B) and can be opened individually. Panels C-G show selected sections that have been opened; these sections have been offset and truncated in this view. See the main text for detailed descriptions of specific sections. Note that FlyBase full gene symbols include a prefix that indicates species, ‘Hsap’ for *Homo sapiens* and ‘Dmel’ for *Drosophila melanogaster*. These are frequently used in the free text portions of the disease reports to avoid confusion because experiments often involve genes from both species.

In September 2015, FlyBase released the first examples of its new integrated reports, the Human Disease Model Reports (Fig. 1). One of the purposes of this new report format is to provide a less specialized entry point for non-*Drosophila* researchers and for *Drosophila* researchers newly interested in a *Drosophila* disease model system. These reports are designed to provide: (1) an integrated presentation of disease-related information from multiple locations within FlyBase; (2) links to other resources, most notably Online Mendelian Inheritance in Man^®^ (OMIM^®^; http://omim.org/); (3) information concerning relationships between predicted human and fly orthologs; (4) a summary of experimental work in *Drosophila* using non-specialist terms; (5) descriptions of experimental work with links to appropriate FlyBase allele records with more detailed information; and (6) listings of relevant genetic reagents. It should be noted that, because FlyBase actively solicits and acts upon input from the user community, the initial version of the Human Disease Model Report described here is likely to evolve over time, depending upon community response and requirements.

As genetic analyses of inherited diseases in humans have become more efficient and robust and the number of causative genes identified has increased, many individual genetic disorders have been redefined as a group of related diseases. For example, the motor neuron disease amyotrophic lateral sclerosis (ALS) currently has over 30 subtypes defined by OMIM, and forms what is termed a phenotypic series. To reflect this, most FlyBase Human Disease Model Reports describe a specific disease subtype, defined by a single causative gene in humans; for example, there are separate reports for ALS1, ALS6 and ALS8, which are subtypes of ALS. These reports include links to a disease report for the ‘parent’ entity (ALS), which describes general and shared information for the phenotypic series. A table showing other members of a phenotypic series, titled ‘Related Diseases’, is displayed in each of the associated parent and subtype reports, as described below.

Fig. 1 provides a visual tour of the Human Disease Model Report for ALS10, the subtype of ALS that is associated with the human gene *TARDBP* (see http://flybase.org/reports/FBhh0000017.html for the full report). Links to OMIM and FlyBase Disease Ontology Term Reports, as well as a link to the parent report for ALS, are provided in the top section (Fig. 1A), followed by an overview that briefly describes the human causative gene, the predicted *Drosophila* ortholog(s) and the types of genetic resources available for manipulation of the fly model (Fig. 1B). The rest of the report initially appears ‘closed’, providing a compact overview that is in line with the standard report organization and presentation design used across FlyBase. Specific sections can be opened one at a time, or the whole report can be opened by using the ‘Open All’ button at the top right. Navigation within a report is facilitated by the floating ‘Report Sections’ panel along the right.

The ‘Disease Summary Information’ section (Fig. 1C) includes background on the human disease, drawn primarily from OMIM. This section includes genetic, cellular, molecular and phenotypic information about the disease and its causative gene(s), and links to further background information. It is followed by the table of ‘Related Diseases’ (Fig. 1D) with links to the relevant OMIM phenotype and gene reports, and FlyBase Human Disease Model Reports; a link to the corresponding OMIM phenotypic series is also provided, immediately above the table. The next section covers orthology (Fig. 1E), identifying the causative human gene (*TARDBP*), and its predicted fly ortholog(s), of which there are two for this example: *TBPH* and *CG7804*. These ortholog predictions are based on the *Drosophila* RNAi Screening Center (DRSC) Integrative Ortholog Prediction Tool (DIOPT), which searches ten ortholog prediction algorithms and displays protein alignments (Hu et al., 2011). A link to the relevant DIOPT page is provided in the next section of the Human Disease Model Report, ‘D. melanogaster Gene Information’. This section also includes molecular function and cellular component Gene Ontology (GO) terms for the fly gene(s), as annotated by FlyBase (Fig. 1F).

The centerpiece of the Human Disease Model Report is the ‘Experimental Findings’ section (Fig. 1G), which presents information from papers that describe results relevant to the disease. The reported information has been sorted into types of experimental data, such as heterologous rescue of *Drosophila* mutants with mammalian transgenes, relevant phenotypes of mutations in *Drosophila* genes, genetic interactions, and drug treatments that have been observed to affect a phenotype; the source publication is provided for each data segment. The ‘Description of Experiments’ subsection shown in Fig. 1G illustrates examples of the information presented. The goal is to make this section accessible to both experienced FlyBase users and to researchers unfamiliar with fly research; thus, descriptions of results are written in a straightforward style with an explicit focus on disease implications. Details of the genetic tools used in the experimental findings are included at the end of each description, with links to relevant FlyBase allele reports.

At the top of the ‘Experimental Findings’ section is a summary that compiles results described in the cited papers into a cohesive story (only a section of the summary is shown in Fig. 1G). The summary discusses the phenotypes resulting from mutations in, RNAi knockdown of, and transgenic overexpression of the disease-associated fly gene, as well as transgenic expression of the human gene in flies, and whether and how these phenotypes recapitulate the human disease. Some models of disease in flies represent phenologs (orthologous phenotypes between organisms that can be identified based on orthology of the underlying genes) (McGary et al., 2010) that correspond to conserved gene networks that have diverged at the level of phenotype. In such cases, the fly phenotypes often bear little resemblance to human disease phenotypes, but functional orthology allows identification of interacting genes, signaling pathways, and biological processes that are impacted by genetic perturbation, as well as of opportunities for drug screening. The summary subsection will be updated regularly, as new findings are incorporated; it will highlight striking results and integrate evolving themes, such as the association of stress granules with neurodegenerative diseases involving RNA-binding proteins (Li et al., 2013) in the report for ALS10 shown as an example here. In the future, the ‘Experimental Findings’ summary section will be followed by a link to a FlyBase Wiki page specific to the disease that is the subject of the report; the aim is to encourage community contributions and comments, particularly concerning this summary section.

The final components of the Human Disease Model Report consist of tabulated presentations of disease-related information from other areas of FlyBase. These include physical interactions involving the orthologous fly gene; alleles of the causative human gene and its predicted fly ortholog that have been annotated with Disease Ontology terms (as described in the following section); and genetic reagents and fly stocks identified as being useful to generate and characterize the fly models of disease.

## Human disease model data in FlyBase Gene Reports

FlyBase Gene Reports include extensive amounts of data that are relevant to models of human disease, including predicted human orthologs of fly genes, experimental results using human genes introduced into flies, descriptions of disease-related phenotypes, and genetic interactions that modify such disease-related phenotypes. FlyBase captures as much information as possible using controlled vocabularies or ‘ontologies’, which allow robust searches and facile query tools. Phenotypes that recapitulate aspects of human disease are annotated using the Human Disease Ontology (DO; http://www.disease-ontology.org) (Kibbe et al., 2015) resource, a controlled vocabulary that contains standardized terms and synonyms for many human diseases. DO is under active development; new terms and relationships between terms are being added during regular updates. The vocabulary is structured as a hierarchical tree with related specific diseases grouped under less specific ‘parent’ terms. For example, individual variants of ALS, such as ALS10, are listed under the more general amyotrophic lateral sclerosis term, which itself is a type of ‘motor neuron disease’ (see the description of ‘Spanning Tree’ below).

**Fig. 2.**
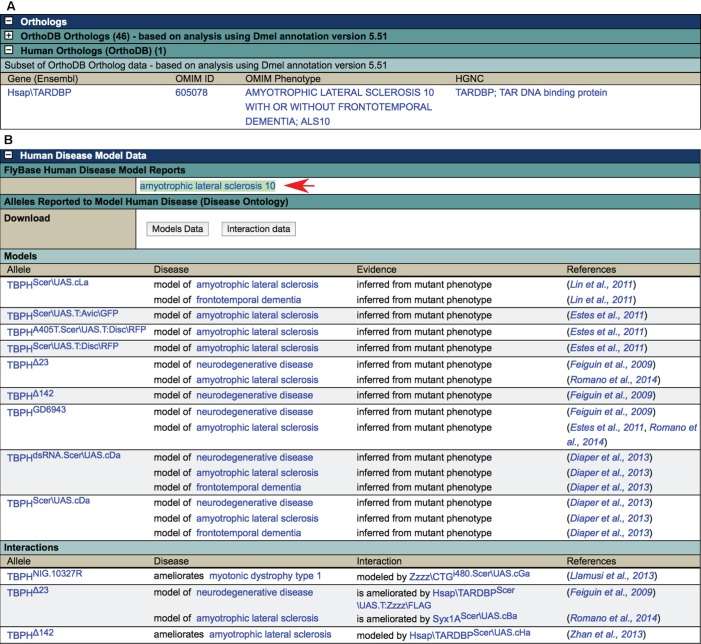
**Key disease-relevant sections in FlyBase Gene Reports.** Sections of the Gene Report for the *D. melanogaster TBPH* gene are shown. The ‘Human Orthologs’ table (A) contains a list of the orthologous human gene(s) as computed by the OrthoDB database. Links to the OMIM and HUGO Gene Nomenclature Committee (HGNC) (Gray et al., 2015) entries for each human gene are provided along with links to the OMIM phenotype descriptions of disease(s) that the human gene has been implicated in. These links are computed from information downloaded from the HGNC and OMIM databases. The ‘Human Disease Model Data’ section (B) reports published fly models of human disease (‘Models’ table), and alleles that have been shown to modify the phenotype of these models (‘Interactions’ table). In addition, a link to the relevant FlyBase integrated Human Disease Model Report is provided (arrow) where one exists. Clicking on an allele symbol takes the user to the appropriate Allele Report, where a more detailed description of the phenotype and a list of available stocks can be found. Clicking on a disease name takes the user to a Term Report for that disease.

FlyBase has extended the Gene Report and Allele Report formats to include any genes, including human genes, introduced as transgenic constructs into flies. Because data capture and queries use existing gene and allele database structures, users see a familiar format when entering a gene report for a human gene and can query across all types of transgenic and endogenous gene data. There are links from genes and alleles mentioned in the Human Disease Model Reports to these more detailed reports. Fig. 2 shows the two key sections in the Gene Report that display human disease information: ‘Orthologs’ and ‘Human Disease Model Data’.

In the ‘Orthologs’ section of *D. melanogaster* Gene Reports, the ‘Human Orthologs’ table (Fig. 2A) lists the predicted human ortholog(s) of the gene, as computed by the OrthoDB database (Waterhouse et al., 2013). For each human ortholog, any OMIM ‘phenotype’ (disease) reports associated with that gene are provided, so that a user can easily see which human disease(s) a *Drosophila* gene of interest might be a candidate to model, based on its orthology. Clicking on the OMIM phenotype name takes the user to the relevant OMIM page, allowing access to detailed information about that human disease.

The ‘Human Disease Model Data’ section (Fig. 2B) shows, first, a link to any Human Disease Model Report associated with the gene. This is followed by an extensive section, ‘Alleles Reported to Model Human Disease (Disease Ontology)’, that reports alleles that have been described as disease models in the published literature, focusing on phenotype-based models. The fly lines used as models fall into three broad categories: ‘classical’ at-locus mutant alleles of *Drosophila* genes, transgenic flies carrying constructs containing a *Drosophila* gene, or transgenic flies carrying constructs expressing a human gene implicated in disease. All of these categories are treated similarly in the database, with an allele created in each case to record and display the relevant information. Alleles whose phenotype recapitulates one or more aspect of a human disease phenotype are labeled in the database with the appropriate disease term from the DO. This information is presented in the ‘Models’ table within the ‘Alleles Reported to Model Human Disease (Disease Ontology)’ section. Below this, in the ‘Interactions’ table, alleles that interact with the established *Drosophila* disease models are shown, listing the interacting allele(s) and how they modify (‘exacerbate’ or ‘ameliorate’) the phenotype of the indicated disease model. A list of the source references is shown next to each disease model or interaction statement. In both tables, clicking on an allele symbol takes the user to the relevant Allele Report, where more detailed information, including a molecular description of the mutant allele or transgene, detailed phenotypic descriptions and availability of fly stocks, is provided.

The phenotypes that FlyBase takes into account when considering whether a potential model recapitulates a human disease phenotype can range from broad behavioral defects or abnormalities at the anatomical level down to changes in molecular properties of cells or gene products. In many cases, the equivalence between the phenotype of the fly model and the human disease is clear, either because a similar cell type is affected or because similar behavioral or physiological defects are observed. For example, the human *NDUFAF6* gene has been implicated in Leigh syndrome (Pagliarini et al., 2008), a severe neurometabolic disorder that arises early in life. Loss-of-function mutations of *sicily*, the fly ortholog of *NDUFAF6*, result in progressive neurodegeneration, impairment of mitochondrial complex I function and increased production of reactive oxygen species (Zhang et al., 2013), symptoms that are all seen in individuals with Leigh syndrome. In a second example, viable missense alleles of *haywire*, the *Drosophila* ortholog of *ERCC3*, lead to ultraviolet sensitivity, modeling one of the hallmark phenotypes of the rare genetic disease xeroderma pigmentosum (Mounkes et al., 1992). Where mutant alleles or transgenic constructs of a single gene have been used to model different diseases, all the diseases are listed in the ‘Alleles Reported to Model Human Disease (Disease Ontology)’ section. For example, the FlyBase report for the human *MAPT* gene shows that transgenic flies expressing human *MAPT* have been used to model several different diseases in which involvement of this human gene is implicated (OMIM; http://www.omim.org/entry/157140), namely frontotemporal dementia, Parkinson disease and Alzheimer disease.

In some cases, the phenotype of a fly line reported as a model for a particular human disease manifests in a tissue that is structurally very different from the tissues affected in humans with the disease. If the fly tissue has been shown to perform functions similar to that of the human tissue, then this is considered to be a recapitulation of the human disease phenotype and the allele is recorded as a model for that disease in FlyBase. For example, loss of function of either the α or β subunit of the *Drosophila* mitochondrial trifunctional protein (Mtp) results in accumulation of lipid droplets in the fat body (Kishita et al., 2012), one of two fly tissues that perform similar functions to the human liver (Søndergaard, 1993). Thus, FlyBase reports this as a model for fatty liver disease, a condition in which excess triglycerides accumulate as lipid droplets in the liver.

In practice, these broad rules for phenotypic equivalence mean that the DO term that FlyBase uses to report the disease model usually reflects what the authors state in the published paper. In rare cases, the fly phenotypes studied appear to be unrelated to the symptoms of the disease that the authors state as being modeled. In this instance, FlyBase does not report the allele as a model of that specific disease but, where possible, a less specific term from near the top of the DO hierarchy, such as ‘neurodegenerative disease’, is used to capture the broad aspect of what is being studied. FlyBase also makes use of these more general terms to capture information for alleles used to model more general processes that might be relevant to several different human diseases, such as metastasis.

There are cases in which an allele might be expected to model a disease or interact with a disease model, but does not. FlyBase records these unexpectedly negative results by indicating that the allele ‘DOES NOT’ model the disease. For example, although the substitutions G328E and R275W in the human *PARK2* gene have both been identified in individuals with Parkinson disease (West et al., 2002; Lücking et al., 2000), only the R275W form results in progressive degeneration of dopaminergic neurons when expressed in transgenic flies (Wang et al., 2007). The gene page for the human *PARK2* gene thus reports that the G328E transgenic allele does not model Parkinson disease. More information describing the experiments performed to reach this negative conclusion, including details of the phenotypes analyzed, are available to users on the relevant Allele Report, which can be accessed by clicking on the allele symbol in the ‘DOES NOT model’ disease statement.

## Querying FlyBase for disease model data: vocabulary Term Reports and QuickSearch

**Fig. 3.**
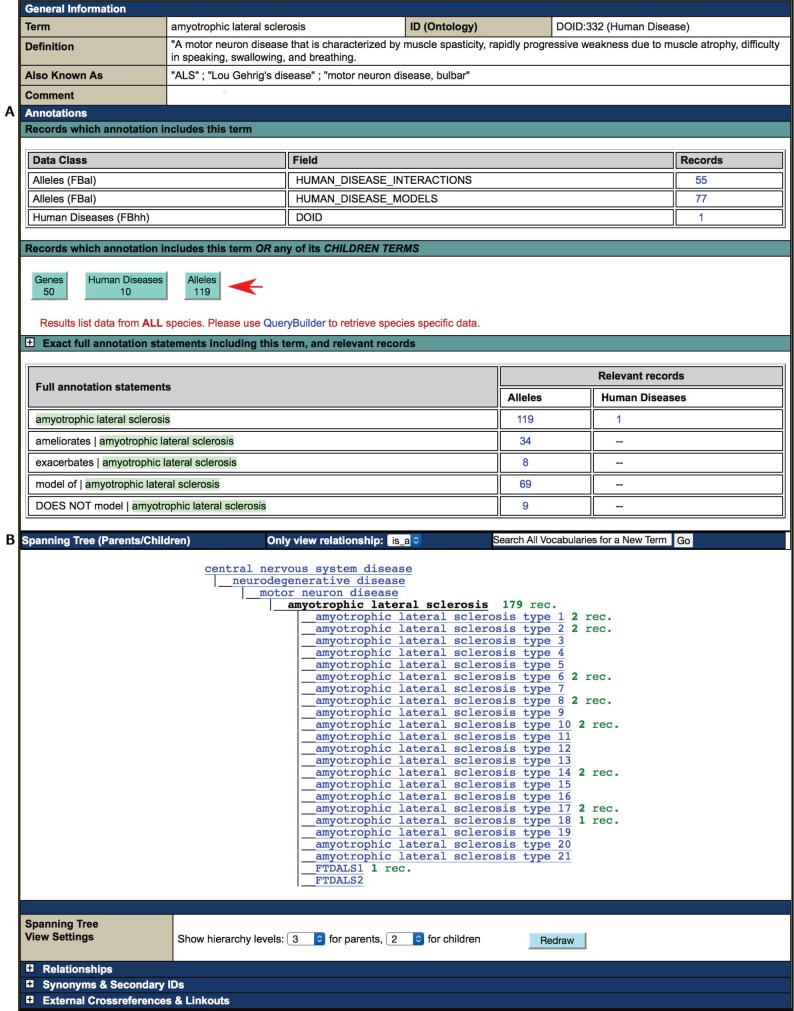
**The Term Report for amyotrophic lateral sclerosis (ALS), based on the Disease Ontology (DO).** The ‘Annotations’ section (A) provides links to genes and alleles that have been used to model the disease or interact with a model of this disease, and to FlyBase Human Disease Model Reports associated with this term. Users can choose to retrieve genes, alleles or diseases labeled with this disease term plus any of its ‘children’ terms (arrowed), or if they are interested only in data labeled with this exact term, they can click on one of the links in the ‘Data Class’ or ‘Full annotation statements’ tables. In each case, the links take the user to a hit list of the relevant genes, alleles or diseases, from which more detailed information can be obtained. The ‘Spanning Tree’ (B) indicates the position of the term in the DO hierarchy, and can be used to explore related disease terms. The current term is highlighted in black and the number to the right of it in green indicates the number of genes, alleles and diseases annotated with that term or one of its children (rec.=records).

Clicking on a disease term within the ‘Human Disease Model Data’ section of a Gene Report or from one of the ‘Disease Ontology ID’ fields in a Human Disease Model Report takes the user to the vocabulary ‘Term Report’ page for that disease (Fig. 3). This page, which is based on the DO, is multifaceted: it provides information and links, hierarchy navigation options and query options. At the top of the page is a brief description of the disease, together with the most common synonyms. The ‘Annotations’ section (Fig. 3A) that follows provides links to hit lists of genes, alleles or diseases that are associated with the disease term in FlyBase, allowing the user to retrieve information that is either linked to the exact disease term, or to the disease term plus any of its more specific ‘children’ terms. The ‘Spanning Tree’ (Fig. 3B) shows the position of the disease term in the DO hierarchy, with the current term highlighted in black and options to move up and down the hierarchy. Using a controlled vocabulary means that all alleles that model a particular disease are labeled with the same term in the database, making it easy to provide links to all alleles and genes involved in a particular disease; the same terms are linked to the corresponding Human Disease Model Reports, providing ready access to all relevant reports in FlyBase.

On the FlyBase homepage (http://flybase.org/), there are several query options that direct users to Human Disease Model Reports and Gene Reports with human disease information. The Term Report pages described above can be accessed directly via the ‘Vocabularies’ tool link, which is also accessible from the ‘Tools’ menu on the top navigation bar. This query tool uses an autocomplete string expansion function, which allows rapid identification of a desired term. If a wide search of many FlyBase data types (including references, genes, alleles and diseases) is desired, the ‘Simple’ search option of the homepage QuickSearch is an all-text search that queries across all types of reports. Another option, the ‘Data Class’ search, allows more targeted searches confined to a single data class and allows (but does not require) searches to be constrained to symbols, names and their synonyms. For all types of data classes, FlyBase aims to capture any synonyms used, which makes such searches more flexible and more useful.

A dedicated tool for disease-related queries is currently under development. This will be an expanded version of the ‘Human Disease’ query tab in the QuickSearch box on the homepage. Input options will include human disease terms and synonyms, human gene designations and synonyms, and *Drosophila* gene symbols, names and synonyms. In addition to relevant Human Disease Model Reports, output will include entry points into the ‘Vocabularies’ pages for the DO. As described above, vocabulary Term Report pages include links to all genes, alleles and diseases associated with a given term.

The FB2015_04 FlyBase release (September 2015) contains an initial set of 44 integrated Human Disease Model Reports, including eight different phenotypic series. In the Gene Reports in this release, DO-based information attributed to over 700 references is available for 177 different human diseases; alleles from 398 genes are reported as disease models, representing 305 *Drosophila* genes and 70 human genes; and alleles from 825 genes are listed as modifiers of a disease. The members of the FlyBase Consortium hope that the two approaches taken to present and integrate human disease model data will enable researchers to easily access this wealth of information and will help them to further harness the extraordinary potential of *Drosophila* for translational research.
